# Supplementary material for: Are carbon emissions trading and green financial instruments synergistic? -Comprehensive quantitative research based on content analysis
Source: PLoS One. 2024 Mar 7;19(3):e0298601. doi: 10.1371/journal.pone.0298601 (PMC10919601; doi:10.1371/journal.pone.0298601)
Supplement: S1 Data — (ZIP) [file pone.0298601.s001.zip › Data/地级市Z_F工作报告.zip!/║■▒▒/╥╦▓2╩╨/└┤╫╘╣1⁄2╓┌║┼í╛┬φ┐╦╩2╛▌═°í┐_2019.docx]

各位代表：

　　现在，我代表市人民政府向大会报告工作，请予审议，并请各位市政协委员和列席人员提出意见。

　　一、2018年工作回顾

　　刚刚过去的一年，是我市改革发展进程中具有重要里程碑意义的一年，必将载入宜昌发展史册。4月24日，习近平总书记考察长江、视察湖北，首站到宜昌，对我市破解“化工围江”、推进新旧动能转换、培育生态小公民等做法给予充分肯定，为宜昌改革发展指明前进方向、赋予新的使命、注入强大动力。总书记的谆谆教导、巨大关怀，令全市上下倍感喜悦和振奋，备受鼓舞与鞭策！乘着总书记视察的浩荡东风，市委六届七次全会确立了“两个走在前列”的奋斗目标，开启了新时代宜昌高质量发展新征程。

　　一年来，在市委正确领导下，我们牢记总书记殷殷嘱托，感恩奋进，真抓实干，埋头苦干，积极应对稳中有变新形势，加快实施“双核驱动、多点支撑、协同发展”战略，始终以第一力度狠抓第一要务，圆满完成市六届人大三次会议确定的各项目标任务，实现了高质量发展良好开局。

　　——经济运行稳中向好。主要经济指标低开高走、全面回升，多项指标达到或超过全省平均水平，完成情况好于预期，扭转了增速下滑的阶段性被动局面。生产总值达到4064.18亿元，增长7.7%；规模工业增加值增长8.5%；固定资产投资增长11.3%；社会消费品零售总额增长11.6%；外贸出口增长9.9%。

　　——生态环境持续改善。城区环境空气质量优良天数达到274天，同比增加16天；PM10、PM2.5浓度分别下降12.5%、8.6%。国考、省考断面水质保持优良。腾退修复长江岸线81.5公里。生态复绿3.95万亩。顺利通过国家森林城市复查。成功获批国家山水林田湖草生态保护修复试点。地方立法、综合执法、生态补偿、智慧治河等生态环境综合治理经验全省推广，全省长江大保护十大标志性战役现场推进会在宜召开，生态保护工作受到省政府重奖。

　　——转型升级步伐加快。强力推进沿江134家化工企业“关改搬转”，新关停企业5家、累计达到30家，改造升级17家、搬迁入园2家、转产3家，精细化工占比提高到33%。压减磷矿开采量300万吨。现有38处煤矿全部关闭退出。工业技改投资增长33%，占工业投资比重57.5%。服务业增加值增长8.5%，占生产总值比重提高到37%。高新技术产业增加值增长13.5%。税收占一般公共预算收入比重70%。单位生产总值能耗下降3.8%、地耗下降5%、水耗下降7%。

　　——民生福祉不断增进。预计城乡居民可支配收入分别增长7.8%、8%，跑赢了经济增速。10.2万人脱贫，55个村出列，远安退出贫困县行列，秭归、兴山有望摘帽。完成易地扶贫搬迁6090户、“消危减土”6.1万户。巩固提升饮水安全52.94万人。新增城镇就业9.7万人。基本养老、基本医保、社会救助等标准稳步提高。棚改新开工1.46万套，基本建成3.12万套。城区改造提升老旧小区66个，新完成畅通工程项目8个，新增公共停车泊位2200个，新建菜市场10个。扫黑除恶专项斗争首战告捷。安全生产“四项指标”大幅下降。

　　一年来，我们对标高质量发展要求，主要做了以下工作：

　　一是精准施策，全力以赴稳增长。面对“三期叠加”特殊困难，针对性实施“三个对冲”举措，及时出台深化工业技改、扶持仿制药发展、壮大现代服务业、激励企业进规进限、加快新旧动能转换、优化营商环境、构建新型政商关系等政策措施，扎实开展“千名干部进千企”服务活动，市场信心有效提振，经济运行回归合理区间。新增市场主体6.32万家、规模以上工业企业96家、限额以上商贸企业299家。运用财政扶持、金融信贷、直接融资等方式，大力支持实体经济发展，金融机构贷款余额达到3105亿元、净增360亿元，新增直接融资180亿元。奥美医疗成功过会，即将上市。着力防范化解重大风险，非法集资陈案化解、新发案件均下降50%以上，金融机构不良贷款率降至1.4%；宜化集团债务化解工作，得到国家银保监会充分肯定。政府债务管理全面加强。

　　二是紧盯项目，全力以赴扩投资。优化招商引资和重点产业项目建设专项考核办法，持续传导压力，以优质项目夯实高质量发展“底盘”。组建14个专业招商专班，分领域分产业推进专业化招商。新开工亿元以上招商项目429个，实际到位资金1675亿元。坚持每月举行重大项目集中开工活动，广汽乘用车生产基地、三宁化工搬迁转型、东阳光原料药等产业项目加快推进；伍家岗长江大桥、白洋长江大桥、秭归长江大桥、江北翻坝高速公路等基础设施项目加快建设；贝因美婴童食品一期、南玻光电玻璃升级等续建项目竣工投产。加大项目谋划争取力度，一批打基础、利长远的重大项目实现突破。宜昌至郑万高铁联络线前期工作加快推进，汉宜高速收费站东迁工程开工建设，页岩气勘探开发取得重大进展。争取中央及省各类转移支付资金超过230亿元。落实对口支援资金141.5亿元。着力激发社会资本活力，民间投资占固定资产投资比重达到63%，5个PPP项目被财政部列为示范项目。

　　三是提质增效，全力以赴转动能。加快发展先进制造业。实施工业技改三年行动，技改面扩大到34.1%。生物医药、电子信息、新材料、装备制造四大新兴产业产值占工业总产值比重38.1%，提高1.5个百分点。12个产业入选全省重点成长型产业集群。启动仿制药一致性评价，完成上报评审品种11个，占全省在审品种三分之二。兴发集团荣获中国工业大奖表彰奖，安琪酵母、人福药业成为全国制造业单项冠军示范企业，奥美医疗成为国家智能制造示范企业。宜都、枝江专业化工园区建设顺利推进。建筑业产值突破1000亿元大关。深入推进农业供给侧结构性改革。柑橘、茶叶等特色产业品种改良达到15.5万亩，绿色生态种植、养殖面积达到4.5万亩，生态养殖畜牧产品产量占比达到25%，“三品一标”新增89个，特色农业产值占比超过85%。加快发展旅游、物流等现代服务业。接待游客7738.2万人次，实现旅游综合收入869.3亿元，分别增长16.9%、21.9%。平湖半岛征迁取得重大突破，三峡国际游轮中心开工建设。枝江东方年华、同心花海，宜都青林古镇，兴山昭君文博园，五峰国际滑雪场等投入运营，一批精品民宿、自驾车房车露营地相继建成。新增4A级景区2家。三峡枢纽水铁公多式联运项目被列为全国示范。商品车滚装翻坝运输正式启动。新增A级物流企业14家，达到70家。电商交易额达到1530亿元，增长23%。荣获“中国美食之都”称号。全面激发“双创”活力，净增高新技术企业55家、高新技术产品210个。新增省级以上孵化器、众创空间7家，城东U谷、三峡创谷等蓬勃发展。来宜就业创业大学生3.3万人。市生物技术、仿制药技术创新公共服务平台挂牌运行。完成科技成果转化运用项目125项。人福药业“药剂高效分装成套设备及产业化项目”获国家科技进步二等奖。万人发明专利拥有量7.26件。新增中国驰名商标2件。安琪酵母获中国质量奖提名奖，市中心医院获长江质量奖提名奖。市委党校迁建工程荣获“鲁班奖”。宜都被列为首批国家创新型县市。

　　四是改革开放，全力以赴激活力。“四多合一”经验全省推广，企业投资项目审批告知承诺、标准地出让、先建后验、局长驻窗等改革成效初显，政务服务“一网覆盖、五级联通”，“3550”目标如期实现。行政审批中介服务管理进一步加强。农村土地确权登记颁证、国有林场改革、事业单位公务用车改革基本完成。县域医共体建设在全省率先实现全覆盖，城区公立医院薪酬制度改革试点启动，枝江公立医院综合改革获国务院表彰。国企、供销、增量配电、综合行政执法、居家和社区养老服务等各项改革有序推进。中央在宜企业“三供一业”移交进程加快。宜昌自贸片区新入驻企业1333家，形成自主创新成果83项、全省复制推广13项。三峡保税物流中心监管货值15.04亿元，增长190%。宜昌综合保税区建设加快。三峡机场旅客年吞吐量达到295万人次，其中出入境旅客5.5万人次。开行中欧（宜昌）班列59列、水运始发班轮516航次，成功开辟铁海联运南向新通道。口岸整体通关时间缩短三分之一。新增外贸备案企业147家。与“一带一路”沿线国家和地区贸易额达到13.8亿美元。成功组织柑橘、茶叶国际采购商“宜昌行”，组团参加首届中国国际进口博览会、外交部湖北全球推介会、鄂港澳粤及日韩经贸洽谈等活动，圆满完成加蓬、几内亚两国元首等外事接待任务，对外开放合作领域不断拓展，城市影响力进一步提升。

　　五是绿色发展，全力以赴优生态。宜昌长江生态保护与绿色发展战略规划加快实施。扎实推进中央环保督察反馈问题整改，顺利迎接“回头看”。领导干部自然资源资产审计有序开展。推进重点工业企业削减31.5万吨标准煤。改造、停用20蒸吨/时及以上燃煤锅炉28台。城区在建工程基本实现扬尘智能监测预警全覆盖。取缔非法码头65个，规范提升码头55个。16个省级以上工业园区污水集中处理设施全部建成，32个城镇污水处理厂完成提标改造，51个新建乡镇污水处理厂达到或基本达到通水条件。城区不达标水体基本消除。与三峡集团合作的长江经济带绿色发展示范项目顺利推进。城区污泥干化处置项目建成运行。“河湖长制”更加完善，“山长制”全面推行。长江岸线宜昌城区段生态保护修复项目全面启动。磨基山公园、求雨台公园等生态园林项目建设进程加快。新建城市绿道20公里、“美丽宜道”187公里。新增绿色建筑228万平方米、装配式建筑32万平方米。71个港口岸电工程建设加快推进。天然气用户增长9.39%。秸秆综合利用率、还田率分别达到94.4%和65.8%。率先在省内推行用能权有偿使用。城区建成生活垃圾分类示范社区50个。改造城区公厕44个、农村厕所10.4万个。出台《宜昌市禁止违法建设管理办法》，城区依法拆违26.9万平方米。宜都被评为国家森林城市。5个镇被列为省级森林城镇，88个村被列为省级绿色乡村，7个村被列入中国传统村落名录。

　　六是共建共享，全力以赴惠民生。财政支出用于民生的比重达到81.3%，人民群众获得感幸福感安全感持续增强。加快补齐交通短板，西陵二路快速路、夷桥路改造、点军大道二期、合益路延伸段、沿江大道下穿通道等建成通车，峡州大道二期、花溪路等加快建设，城区快速路网内环实现闭合、中环顺利推进、外环基本成型。市域城际公交在全省率先实现全覆盖。建成一级公路57公里、二级公路233公里，硬化农村公路1958公里，基本完成通客班车村道安防工程。累计完成228个贫困村电网改造，农网户均容量提前达到国家标准。完成天然气调峰储备一期工程。新建、改扩建幼儿园20所，公办和普惠率达到77.8%。“择校热”“大班额”现象得到有效遏制。高中阶段教育结构性改革获全国教育改革创新奖。特殊教育、职业教育、高等教育等各级各类教育协调发展。市青少年实践教育基地被列为国家研学示范营地。坚持“塑形、铸魂、惠民”，社会主义核心价值观广泛弘扬，群众性精神文明建设成效显著，顺利通过全国文明城市年度测评、国家公共文化服务体系示范区创建验收。市中心医院综合大楼、市三医院结核病防治大楼、市儿童医院一期等加快建设。基层医疗机构服务能力不断增强。实现市域内异地就医“一站式”结算。西陵区被列为国家健康促进区。成功举办屈原故里端午文化节、第28届中国厨师节、首届农民丰收节、宜昌国际马拉松赛等节赛活动。成功申办2022年省运会。“雪亮工程”经验全国推广，“七五”普法中期检查顺利通过，平安宜昌、法治宜昌建设取得新成效。村（居）民委员会换届全部完成，基层自治能力有效提高。残疾人服务保障更加有力。全社会支持参与慈善事业的氛围更加浓厚。

　　一年来，我们始终把党的领导贯穿于政府工作全过程，着力锻造“严紧硬实”作风，行政效能明显提升，政府自身建设全面加强。深化完善党的建设、业务工作、安全生产“三同步”责任区工作制度，严格履行全面从严治党主体责任。认真落实中央八项规定精神和省委、市委实施意见，加强政府系统廉政建设和反腐败斗争。完善政府工作规则，健全议事决策规范，强化决策合法性审查。自觉接受人大及其常委会、政协、社会舆论等监督。实现乡镇扶贫审计全覆盖。提请市人大常委会审议地方性法规2部，办理人大代表议案和建议291件、政协委员建议案和提案426件。与工会、共青团、妇联等群团组织联系更加紧密。

　　国防动员、人民防空、民兵预备役、退役军人、双拥等工作进一步加强。援藏援疆、民族宗教、对台、信访、档案、保密、方志、科普、气象、消防、老龄、防震减灾、新闻出版、广播电视、政策咨询、哲学社会科学等工作取得新成绩。

　　各位代表！成如容易却艰辛。过去一年，稳增长难度之大、风险挑战之多前所未有。面对巨大压力，全市上下众志成城、攻坚克难，经受了重大考验、稳定了发展大局、推动了转型升级、守住了红线底线，特别是解决了一些长期想解决而没有解决的难题、办成了一批过去想办而没有办成的大事，宜昌发展迈上新台阶、呈现新气象。这是习近平新时代中国特色社会主义思想科学指引的结果，是省委省政府和市委坚强领导的结果，是市人大、市政协监督支持的结果，更是全市广大干部群众实干拼搏的结果。在此，我代表市人民政府，向全市人民，向各位人大代表、政协委员，向各民主党派、各人民团体和各界人士，向中央、省属在宜单位，向驻宜部队、武警官兵，向所有关心支持宜昌改革发展的海内外朋友，表示衷心的感谢！

　　各位代表！安不忘危、兴不忘忧。我们清醒认识到，发展中还存在一些不平衡不充分的问题：化工等传统产业转型升级任务繁重，新经济培育不够充分，现代服务业发展还有很大空间；重大产业项目偏少，高质量项目不多，区域发展分化明显；科技创新能力较弱；教育、医疗、养老、住房、生态环保、交通出行等方面，群众还有不满意的地方；表态多调门高、行动少落实差在少数地方和部门还不同程度存在。我们一定正视问题、直面矛盾，采取有效措施，切实加以解决，决不辜负人民重托！

　　二、2019年工作重点

　　各位代表！新的一年孕育新的力量，新的起点昭示新的希望。今年是新中国70华诞大庆之年，是决战决胜全面建成小康社会的关键之年。非常之年须有非常作为，我们必须以新业绩回应人民群众新期盼。根据市委六届九次全会部署，我们将以习近平新时代中国特色社会主义思想和党的十九大精神为指导，深入落实习近平总书记视察湖北重要讲话精神，对标新发展理念和高质量发展，坚持稳中求进工作总基调，全面落实“六稳”要求，以供给侧结构性改革为主线，以深化市场化改革和扩大高水平开放为动力，认真落实省委省政府“一芯两带三区”战略布局，以“增动能、提效能”为抓手，深入实施“双核驱动、多点支撑、协同发展”战略，统筹推进“稳促调惠防”各项工作，继续打好“三大攻坚战”，促进经济平稳健康发展、社会大局和谐稳定，加快实现“两个走在前列”奋斗目标，为全面建成小康社会收官打下决定性基础，以优异成绩迎接新中国成立70周年。

　　今年的主要预期目标是：生产总值增长7.5%以上，规模工业增加值增长7.5%以上，固定资产投资增长11%以上，社会消费品零售总额增长10.5%以上，外贸进出口总额增长6%，居民收入增长高于经济增速，全面完成省政府下达的节能减排和环境保护任务。

　　同时，还应努力实现以下目标：服务业增加值占生产总值比重提高到38.5%，研发与试验发展经费支出占生产总值比重达到2%，税收占一般公共预算收入比重超过70%，规模工业增加值能耗下降7.9%，城区空气质量优良天数比例77%，地表水达到或好于Ⅲ类水体比例88.9%。

　　提出上述目标，目的是引导全市上下把发展的注意力、着力点转到提高质量效益上来。高质量发展既要重质量，也要讲总量。质量提不高，竞争力上不来，走不到前列；速度跟不上，总量做不大，也难以发挥省域副中心城市辐射带动作用。我们将坚定不移落实稳中求进工作总基调，该稳的坚决稳住，该进的积极进取，实现经济平稳增长和提高质量效益互促共进；坚定不移把改革进行到底、把开放引向深入，不断提升改革开放水平；坚定不移践行习近平生态文明思想，坚持生态优先，加快绿色发展；坚定不移以人民为中心，努力增进民生福祉，切实把总书记的关怀厚爱、殷切期望转化为推动宜昌高质量发展的实际行动。

　　今年，我们将重点做好七个方面工作：

　　（一）更大力度提升改革开放水平。改革开放是事业发展进步的活力之源和重要法宝。我们将深入解放思想，乘势而上，改革不停顿，开放不止步，全力推动新时代改革开放再出发。

　　深化重点领域改革。按照“巩固、增强、提升、畅通”要求，加快供给侧结构性改革。纵深推进“去降补”，依法处置“僵尸企业”，坚决淘汰落后产能，降低企业用能、物流、制度性交易成本。优化政府机构设置和职能配置，按期完成政府机构改革任务。推动公共资源交易全程电子化管理。加快投融资体制改革，规范推进PPP项目。强力推进国企国资改革，严格国资监管。加快国有投融资公司市场化、专业化转型。加强国有企业现代企业制度建设。整合市属国有港口资源，组建市级港口运营平台。推动宜粮集团实质性运作。健全财政预算绩效管理制度。深化税收征管体制改革。强化“双随机、一公开”监管。抓好增量配电、居家和社区养老服务、经济发达镇行政管理体制等改革试点任务。积极推进流域治理、城市管理领域和县市区、乡镇综合执法改革。稳步推进农村土地所有权、承包权、经营权分置改革。完成农村集体产权制度改革试点任务。加快供销社综合改革。深化医药卫生体制改革。推进生态环境损害赔偿制度改革。制定实施高质量发展评价与考核体系。

　　推动形成全面开放新格局。全面完成自贸片区改革试点任务，再释放一批制度创新红利。积极推进跨境电商产业园建设，力争国际铁路陆港产业园、汽车平行进口展销中心、进口商品交易中心等项目落地建设。深度融入“一带一路”，着力打造大通道、大通关、大平台。加快综合保税区申报建设，确保年内达到封关运营条件。支持三峡保税物流中心申报省级外贸综合服务试点、三峡物流园申报国家市场采购贸易方式试点。加快实施三峡机场二期改扩建项目。力争三峡机场口岸扩大开放获批，宜昌港开放纳入国家审理计划。提升中欧（宜昌）班列运行效率，加密水运口岸始发航班。深入实施外贸“三项工程”，确保新增开口企业20家。建好出口农产品示范基地。力争进出口额达到32亿美元。完善政策措施，引导外资更多投向先进制造业、现代服务业、现代农业、生态建设等领域，确保利用外资2.9亿美元。支持企业参加国际国内会展，“走出去”建设境外园区。加强与国际友城交流合作。

　　打造一流营商环境。聚焦“成本最低、效率最高、服务最优”目标，深化“放管服”改革，加快营造省内领先、全国一流营商环境。全面推行“证照分离”“照后减证”，抓好直接取消审批、审批改为备案、优化准入服务等重点工作。全面取消无法律法规依据的证明事项。巩固政务服务“一网覆盖、五级联通”成果，加快政府系统信息互通共享，力争“一窗”分类受理事项达到80%以上，200个以上高频事项实现“最多跑一次”。把企业开办、不动产登记、投资项目开工许可、项目竣工验收等改革成果制度化、常态化。加强市县乡村政务服务体系标准化建设。强化行政审批中介服务管理。加强社会信用体系建设。纵深推进“千名干部进千企”服务活动，重点解决一批行业共性问题。

　　（二）更大力度推动经济稳健增长。“稳”是发展的基石，行稳才能致远，求进才能突破。我们将以进促稳、以稳应变，推动经济在质的提升中实现量的有效增长。

　　积极扩大有效投资。深入开展重大项目“集中开工+集中签约”活动，持续强化开工项目现场督察、投产项目“双月巡礼”，每月进行视频调度，定期通过媒体公布考核结果，推动项目洽谈签约一批、落地建设一批、投产达效一批。加快三宁化工乙二醇、兴发集团转型升级等续建项目建设，推动宜化搬迁入园、东阳光创新药仿制药、三峡卷烟易地搬迁技改等项目尽快开工。加快推进白洋长江大桥、秭归长江大桥、远当地方铁路等项目建设，开工建设宜昌至郑万高铁联络线、呼南高铁宜昌至荆门段、宜张高速渔洋关至鄂湘界段、宜来高速宜昌段等项目，做好城区轨道交通2号线一期、江南翻坝铁路一期、白洋港疏港铁路、当（阳）枝（江）松（滋）高速、襄宜高速、神农架至五峰高速等项目前期工作。建好投资项目储备库、建设库、达效库，确保转化率30%以上。扎实推进产业链招商、专业招商、驻点招商、商会协会招商，确保新开工亿元以上招商项目410个，招商引资到位资金1600亿元。深化与三峡对口支援单位经济协作。

　　培育壮大市场主体。加大企业“进规”“进限”扶持力度，择优遴选500家高成长性小微企业，一企一策，持续帮扶，确保净增规模工业企业、限额商贸企业各100家。引导骨干企业聚焦实业、做精主业，扎根宜昌发展，实现“二次创业”。支持企业向“专精特新”发展，努力培育一批“小巨人”“单项冠军”和“隐形冠军”。力争和远气体成功上市，均瑶大健康完成报会。深化与中省企业战略合作，支持在宜企业发展壮大。完善促进消费的政策和体制机制，统筹做好重点商圈、专业市场、特色商业街区建设，进一步挖掘消费潜力，努力打造鄂西消费中心。大力发展电商产业，力争交易额突破1800亿元。

　　繁荣发展民营经济。坚持“两个毫不动摇”，全面落实国家、省政策和市扶持民营经济发展“二十条”，让民营企业家安心谋发展，确保民间投资占固定资产投资比重达到65%。市财政筹资10亿元支持融资担保体系建设，筹资10亿元设立民营及小微企业票据贴现中心，引导金融机构、小贷公司、风险投资等更好支持民营经济发展。探索建设“宜昌网上金融服务大厅”，收集整合涉企信用数据，推动免抵押担保信用融资。清理、精简涉及民间投资管理的行政审批事项和涉企收费。完善领导干部联系重点民营企业机制，充分发挥非公有制企业投诉服务中心作用。鼓励民营企业参与国有企业改革。依法保护民营企业家人身和财产安全，保障企业合法经营。大力弘扬企业家精神，引导民营企业家争做爱国敬业、守法经营、创业创新、回报社会的典范。

　　各位代表！民营经济是推动经济发展的重要力量。凡是民营企业的合理诉求，我们坚决有求必应；凡是政府作出的承诺，我们坚决兑现落实。真心实意帮扶，真金白银支持，让民营企业和民营企业家有实实在在的获得感、公平感、安全感！

　　（三）更大力度培育新的发展动能。新旧动能转换是高质量发展的内在要求。我们将以动力变革推动质量变革、效率变革，让传统产能焕发生机，让新的动能挑起大梁。

　　强力推进新型工业化。完善政策措施，统筹用好各级传统产业改造升级专项资金。加大工业技改力度，重点围绕智能制造、绿色制造、质量提升谋划项目，确保技改投资800亿元、占工业投资比重超过50%，规模工业企业技改面新增25%以上。推动华强科技、三峡制药硫酸新霉素等255个工业项目建成投产。分类推进沿江化工企业“关改搬转”，依法再关停4家，改造升级18家、搬迁入园14家、转产4家。抢抓园区扩区调规机遇，加快宜都、枝江专业化工园建设。强化协调服务，确保广汽乘用车项目3月份首台整车下线、6月份实现量产，加快培育汽车及零部件产业。积极发展军民融合产业，加快推动710所国家深海智能装备产业园和总部研发基地、航天科工商业火箭发动机总装基地等项目落地建设。力争生物医药、电子信息、新材料、装备制造等新兴产业占工业总产值比重提高1个百分点以上。支持页岩气勘探开发、商业化利用，力争纳入国家页岩气综合开发示范区。实施“两化”融合提升行动，新增国家级贯标认定企业1家、省级试点示范企业15家。

　　促进现代服务业全面升级。推动“大旅游”产业提质增效。加快三峡国际游轮中心建设，深度开发下牢溪旅游片区，推动“两坝一峡”旅游区提档升级。积极创建三峡国家级旅游度假区。推进巴山金谷、清江画廊倒影峡等项目建设，加快伍家岗南灵、点军曹家畈、夷陵三峡茶旅、远安太平顶、长阳火烧坪等“旅游+”特色小镇建设。支持三峡大瀑布、西陵峡旅游区、当阳玉泉风景区创建5A级景区，宜都天龙湾、远安武陵峡、兴山昭君村、长阳卓尔创建4A级景区。推进全域旅游示范创建，促进旅游与文化、农业、体育、研学深度融合。新增4家优品饭店、6家银宿级以上精品民宿。确保全年接待游客数增长15%、旅游总收入增长12%。推动“大物流”产业转型升级。深化与上港集团、招商局集团、民生轮船企业合作，加快构建三峡翻坝转运通道，推进多式联运发展。力争《宜昌港总体规划（修订）》获批实施。促进茅坪港、白洋港稳健运营。大力发展园区物流、专业市场物流、产业集群物流、城乡配送物流，培育壮大一批物流骨干企业，新增A级物流企业15家，物流业增加值增长12%。推动“大健康”产业快速发展。支持企业加快仿制药研发，开展一致性评价，努力打造国内一流仿制药生产基地。加快推进亚行贷款综合养老服务示范、宜都山城水都健康城、兴山榛子高山旅游康养等项目建设。推动“大数据”产业加快成长。全力推进三峡数据存储与灾备中心、紫阳大数据一期等项目建设。谋划建设工业互联网平台，推动1000家企业上云。

　　提升科技创新能力。紧盯短板，持续发力，抓实抓好“科技型中小企业成长工程”“高新技术企业倍增工程”，着力壮大高新技术产业，力争净增高新技术企业100家，高新技术产业增加值占生产总值比重提高2个百分点。依托兴发集团等龙头企业，组建面向行业的技术创新公共服务中心。积极创建国家级、省级研发平台。引导企业加大研发投入。强化中小企业创新支持。支持在宜高校、科研院所参与研发平台和“双创”基地共建。促进科技、产业、投资融合对接，让更多科技成果在宜转化。支持宜昌高新区、西陵区创建“全国双创示范基地”，支持枝江创建国家创新型县市。深化“宜才宜用·双招双引”，建立“资智回宜”长效机制，实施“我选湖北·爱上宜昌”计划，引导更多大学生、更多优秀人才来宜发展，新增2.1万名以上大学生来宜就业创业。实施“千百十”质量强企工程，加快建设质量强市。确保有效注册商标数量持续增长，力争新增地理标志商标5件以上。不断提升三峡检验检测中心区域辐射能力。

　　（四）更大力度打好三大攻坚战。三大攻坚战个个都是硬骨头、硬任务。我们将紧盯年度目标，针对突出问题，以打硬仗的决心、钉钉子的韧劲，尽锐出战，精准施策，强力清障碍、跨关口，为如期全面建成小康社会奠定坚实基础。

　　防范化解重大风险。完善长效机制，强化风险监测和应急处置，做到坚定、可控、有序、适度。深入开展非法集资、互联网金融风险等专项整治，加强类金融机构监管，多措并举化解银行不良贷款，推动地方法人金融机构稳健运营。实施国企负债规模和资产负债率管控约束机制。发挥债权人委员会、应急周转金等作用，妥善处置重点企业债务风险。严格规范政府举债行为，积极稳妥推进存量隐性债务化解工作，用好用活政府新增债券和置换债券等政策，严格将市级政府债务余额控制在限额内。加大风险楼盘处置力度。

　　决战决胜脱贫攻坚。突出“准、实”导向，下足“绣花”功夫，坚持现行标准，确保4万人脱贫、16个贫困村出列、五峰和长阳摘帽，坚决实现基本消除绝对贫困目标。改善深度贫困地区发展条件，完成15个贫困村电网改造任务，“消危减土”1.5万户。强化对老年人、残疾人、重病患者等特殊贫困人口帮扶力度。深化“双建双包双带双促”帮扶行动，落实产业发展指导员制度，提升就业、生态、电商、金融、教育、健康、综合保障等扶贫实效。探索推广慈善扶贫超市、互助养老等扶贫新模式。引导各类企业、社会组织参与脱贫攻坚。坚持扶贫与扶志扶智相结合，激发内生动力，营造勤劳致富、脱贫光荣的浓厚氛围。认真落实中央脱贫攻坚巡视反馈意见。深化扶贫领域腐败和作风问题专项治理，坚决反对弄虚作假、数字脱贫，坚决查处贪污挪用、截留私分、虚报冒领扶贫资金等腐败案件。

　　重拳整治环境污染。以长江大保护十大标志性战役为主抓手，打好蓝天、碧水、净土保卫战。强力推进“散乱污”企业、柴油货车污染、散煤、非道路移动机械等专项治理，扎实开展重点行业挥发性有机物、船舶污染、工地扬尘等综合整治，确保主要环境空气污染物浓度持续下降。加强大气监测设施及能力建设，编制大气污染排放源清单。加快推进城区污水厂网与生态水网共建项目，持续改善城区水环境。确保在建乡镇污水处理设施全部投入运营。推广黄柏河流域治理经验，加强沙河、玛瑙河等河湖综合治理，完成“清四乱”整治任务。实现小微水体“河湖长制”全覆盖。抓好小型水电站生态流量泄放工作。加强农业面源污染治理。开展土壤污染详查。探索建立污染地块名录和开发利用负面清单。加快推进关停化工企业土壤修复治理。持续抓好中央环保督察“回头看”反馈问题整改。坚决制止和惩处破坏生态环境行为。

　　（五）更大力度推进美丽宜昌建设。生态环境是面向未来的最大竞争力。我们将高擎“绿色指挥棒”，守好“绿色责任田”，做好生态修复、环境保护、绿色发展“三篇文章”，留住绿水青山，造就金山银山。

　　抓好生态保护和修复。加快实施宜昌生态文明建设示范市规划，严守资源环境生态红线、永久性基本农田控制线，科学确定城镇开发边界。全面推进山水林田湖草生态保护修复国家试点。统筹实施全域生态复绿六大工程，坚持“四季挖窝、三季种树”，复绿长江干支流岸线5252亩、73公里，公路绿色通道提升造林1768亩、895公里，精准灭荒造林4013亩，复绿关停废弃矿山1156亩、工程临时占地1147亩。继续抓好林业有害生物防治工作。基本完成滨江公园白沙路至柏临河段生态修复，开工建设柏临河入江口至猇亭古战场段生态整治工程。保护修复城区山体300万平方米。实施最严格的饮用水源地保护制度。巩固非法码头治理成果，推进岸线资源清理整顿和无序码头专项整治。规范建设砂石集并中心，严厉打击河道非法采砂。完成三峡坝区岸电实验区建设。加快绿色港口一体化发展。新建城市绿道10公里以上。

　　加快形成绿色生产生活方式。深入推进绿色低碳循环发展，培育壮大节能环保、清洁生产、清洁能源等绿色产业。加大工业园区循环化改造力度，引导企业节能降耗、降本增效。促进餐厨垃圾、建筑垃圾、畜禽养殖废弃物等资源化利用。深化建设用地总量和强度、磷矿开采总量与矿权数量等双控行动。开展“大棚房”问题专项清理整治。推进绿色矿山建设。完善用能权有偿使用机制。加快推行生活垃圾分类，城区新建100个垃圾分类示范社区，公共机构强制分类覆盖率达到60%。大力发展装配式建筑，新增装配式建筑40万平方米。鼓励发展被动式超低能耗建筑。探索扩大磷石膏规模化利用途径。倡导简约适度、绿色低碳生活方式，深入开展绿色家庭、绿色学校、绿色社区创建行动。放大“三峡蚁工”效应，争做“生态好公民”。

　　努力建设生态城市。加强城乡规划设计，编制完成宜昌2050发展战略规划。以城市骨干道路为突破口，实施增色添彩，助推生态提质，加快建设花园城市。坚持节俭原则，突出地方特色，实施峡州大道、城东大道、东山大道、夜明珠路等彩化工程，完成15个花园城市项目。鼓励单位和居民绿植外移、临街亮花。扎实开展城市建设绿色发展三年行动。积极推进海绵城市建设。打通大动脉、疏通微循环，加快建设畅通城市。着力完善城区快速路网，力争建成通车80公里。加快推进伍家岗长江大桥、沿江大道延伸段、峡州大道二期等项目建设，确保汉宜高速收费站东迁、花溪路下穿汉宜高速段、点军大道三期、东山大道延伸段、夷陵区“三路一匝”等上半年建成通车。启动江城大道下段、港窑路延伸段、宜长公路等项目建设。优化城市公交线网。新建公共停车场10个，新增泊位3400个。科学规划高铁北站片区，有序推进共联滨江片区开发，持续推动火车东站、奥体中心、沙唐等片区深度开发。提高城市管理科学化、精细化、智能化水平。城区依法拆违20万平方米，打造无违建小区20个。

　　各位代表！绿水青山，天赋予宜，用之不觉，失之难存。我们将始终秉持生态惠民、生态利民、生态为民理念，以更加坚强的决心、更加有效的举措保护修复生态环境，让蓝天白云常驻、清水绿岸常存、鸟语花香常在，加快建设宜昌人民引以自豪的生态乐园、美丽家园！

　　（六）更大力度促进区域协调发展。区域协调发展是建设现代化经济体系的重要任务。我们将全力打造核心引擎，加强区域联动，促进城乡融合，推动比学赶超，努力形成“双核驱动、多点支撑、协同发展”新格局。

　　切实增强城区经济实力。出台支持城区发展政策，推动经济管理权限下放，进一步扩权强区。

　　加强城区梯度财源建设。优化宜昌高新区体制机制，强力推进沙湾、善溪窑、田家河片区开发，加快建设白洋新城，增强要素聚集、科技创新能力，积极培育工业经济、现代物流等千亿产业，尽快形成高质量发展的领头雁。支持西陵加快发展都市工业、总部楼宇经济，争创全国服务业综合改革试点区。支持伍家岗加快壮大先进装备制造、城市旅游产业，打造都市经济样板区。支持点军加快发展电子信息、文化旅游、康养产业，建设滨江生态新区。支持猇亭加快发展汽车及零部件、精细化工、临空经济产业，构筑产城融合的现代化新城区。支持夷陵多领域对接、全方位融入主城区，加快推动城市空间共管、基础设施共建、产业发展共育、公共服务共享。

　　努力提升县域经济质效。认真落实省委省政府推动县域经济高质量发展意见，坚持全要素、全产业链、全地域谋划布局和发展县域经济。以开发区、园区为依托，引导各县市培育主导产业，形成特色优势，实现错位发展。加快发展“飞地经济”。推动市县产业、项目、基础设施有机对接，鼓励各类要素资源在市域内优化配置。支持宜都、枝江、当阳在全省争先进位，更好发挥示范引领作用。支持远安、兴山、秭归、长阳、五峰竞相发展，加快提升支撑实力。

　　深入实施乡村振兴战略。聚焦“五个振兴”，编制实施宜昌乡村振兴战略规划。开展乡村振兴示范县市、示范乡镇、示范村创建。实施橘园“三改”8万亩，新建无性系茶叶示范园10个，发展绿色生态养殖，启动推广“双水双绿”稻田综合种养模式10万亩，推动特色农业稳规模、优结构、提品质、促融合。因地制宜发展中药材产业。放大“宜昌蜜桔”“宜昌宜红”“宜昌猕猴桃”品牌效应，培育茶叶、高山蔬菜、生态养殖等区域农产品公用品牌。发挥柑橘品种、品牌优势，着力打造三峡“橘谷”，建设国际一流高品质柑橘生态产业带。提升农产品加工业、休闲农业、农村电商、专业合作社、家庭农场、田园综合体等发展水平。大力开展农产品质量安全县市创建。新建一批农产品标准化生产基地。加快实施“三乡工程”，引导资本、人才、技术更多流向农业农村。提高农业科技创新和社会化服务水平。加快实施“一村多名大学生”培养计划。大力培育新型职业农民。学习浙江“千村示范、万村整治”经验，开展以“三清一改”为重点的村庄清洁行动，支持宜都、枝江、远安、夷陵争创全国农村人居环境整治示范县市。完成农村公路提档升级1000公里，实施自然村通畅工程500公里，建成“美丽宜道”150公里。巩固提升农村饮水安全29万人。加强农村公共文化建设。健全乡村治理体系，提升治理能力。

　　各位代表！区域协调发展，既要城市繁荣，也要乡村红火；既要“双核”发力，也要“多点”奋进。我们将凝聚各方力量、统筹要素资源、激发市场活力，再造城区经济新优势，打造县域经济升级版，开创百舸争流、抢前争先的生动局面！

　　（七）更大力度保障和改善民生。人民对美好生活的向往，是我们始终不渝的奋斗目标。我们将始终把群众的安危冷暖、酸甜苦辣放在心上，尽力而为、量力而行，实实在在帮群众解难题、为群众增福祉、让群众享公平。

　　提高就业和社会保障水平。落实国家、省就业创业政策，开展就业提升行动，新增城镇就业7.5万人。大力支持农村劳动力转移就业和农民工返乡创业。着力拓宽城镇就业困难人员、企业分流职工、零就业家庭等群体就业渠道。做好退役军人安置、就业创业和解困帮扶工作。加大企业稳岗、创业担保贷款等支持力度。优化公共就业创业服务。提高就业技能培训实效。推行农民工工资专用账户管理。坚持兜底线、织密网、建机制，加快建设覆盖城乡居民的多层次社保体系。按政策调整提高社保待遇。加强对困难群众、特殊群体的兜底保障。完善社会救助、社会福利、慈善事业、优抚安置等制度。保障妇女儿童、老年人、残疾人合法权益。因城施策、分类指导，加快建立多主体供给、多渠道保障、租购并举的住房制度，促进房地产市场平稳健康发展。开工棚改2696套。城区改造老旧小区100个，新建改造菜市场15个。加快力能天然气调峰储备二期工程建设。确保官庄至梅子垭水库连通工程建成通水。

　　扩大公共服务有效供给。坚持教育优先发展，落实立德树人根本任务，加快发展更高质量更加公平的教育。优化教育支出结构，重点向贫困地区、薄弱环节倾斜。加快实施第三期学前教育行动，新建、改扩建幼儿园12所，下大力提高公办园数量。促进义务教育优质均衡发展，推动区域教育现代化达标县创建。办好优质化、多样化、特色化普通高中。创新发展职业教育，深化产教融合、校企合作，更好服务地方发展。实施第二期特殊教育提升计划。支持三峡大学建设国内一流大学、一流学科，三峡职院创建高水平高职院校、高水平专业。加强校园安全建设。强化校外培训机构治理。完善困难家庭学生资助政策。支持和规范社会力量兴办教育。深化全国健康城市建设，促进医养融合发展，加快提升基本医疗和公共卫生服务保障水平。全面落实异地就医结算政策，扩大定点机构覆盖面。加强全科医生、儿科医生等队伍建设。完善“互联网+医疗卫生”服务。传承发展中医药事业。加快市中心医院综合大楼、市二医院老年病医院等项目建设。落实全面两孩政策，优化配套服务。支持宜都、枝江创建国家卫生城市。确保宜昌国家卫生城市“四连冠”。大力培育践行社会主义核心价值观，深化群众性精神文明创建活动，加快新时代文明实践中心建设，提高公民文明素质和城市文明程度。开展系列文化惠民活动。确保市新博物馆和奥体中心6月份、市妇女儿童活动中心10月份投入使用。办好全国举重锦标赛、第十届长江三峡国际旅游节等节赛活动。有序推进省运会筹备工作。

　　推动社会治理共建共治共享。完善城乡社区服务功能，深化拓展网格化服务管理。抓好信访维稳工作，健全社会矛盾纠纷排查化解机制。加强行政复议、行政应诉及仲裁工作。深化法治宣传教育，提高全民法律素质。加强人民调解工作，提升公共法律服务水平，完善法律援助制度。深入推进扫黑除恶专项斗争，依法打击各类违法犯罪活动。进一步拓展“雪亮工程”应用领域。推动食品安全示范县、示范乡镇、示范村创建。严格落实粮食安全行政首长负责制。加强应急管理，提升防灾减灾救灾能力。强化安全生产风险管控和隐患排查整治，坚决守住安全生产红线。

　　深入开展国防教育和双拥共建，争创双拥模范城“七连冠”，支持驻宜部队改革和现代化建设，巩固发展军政军民团结良好局面。加强统计体系建设，提高统计质量。完成第四次经济普查工作。做好第三次国土调查工作。支持工会、共青团、妇联等群团组织更好发挥作用。

　　三、切实提高政府执行力

　　各位代表！执行力就是政府工作的生命力。我们将始终把提高政府执行力作为政府自身建设的重要着力点，切实做到讲政治、善谋事、会创新、敢担当、能落实，努力建设让党放心、人民满意的务实高效政府。

　　坚持旗帜鲜明讲政治。提高政府执行力，必须把讲政治摆在首要位置、作为根本要求。提高政治站位，树牢“四个意识”，坚定“四个自信”，践行“两个维护”，切实增强落实党中央决策部署的政治自觉、思想自觉、行动自觉，确保政令畅通、令行禁止。始终在市委领导下开展工作，严格执行市人大及其常委会决议决定，依法接受法律监督、工作监督，自觉接受人民政协的民主监督，主动接受社会监督和舆论监督，认真听取民主党派、工商联和各界人士意见，为推动宜昌改革发展凝聚广泛力量！

　　坚持攻坚克难勇担当。敢于担当作为，既是政治品格，也是从政本分。始终把使命和责任放在心上、扛在肩上，坚持以知难而进的勇气、敢闯敢试的胆量、久久为功的韧劲，勇挑重担子，敢啃硬骨头，在积极应对重大挑战、抵御重大风险、克服重大阻力、解决重大矛盾中彰显担当、展现作为，努力让各项工作不负时代、不负人民、不负历史。不断创新工作思路、机制和方法，着力从困境中探索出路、找到办法、办成事情，确保执行力度更大、效果更好。强化正向激励，切实为担当者担当、为负责者负责，让干事创业者有定力而心无旁骛、有动力而义无反顾！

　　坚持真抓实干重落实。为政之要，重在实干，贵在落实。大兴学习之风、调研之风、落实之风，践行“一线工作法”，主动在改革发展最前沿发现问题、推动工作，在民生改善第一线掌握诉求、解决矛盾，确保事关发展的要事、民生的大事落细落实落地。强化“五个过硬”，增强“八大本领”，善于运用新的理念、法治思维和市场办法谋发展、惠民生，争做“工匠+猛将”型干部。深入推进政府效能建设，切实做到“文不过夜、事不隔天”“事事有着落、件件有回音”，坚持说了就办、定了就干、干就干好，打造落实闭环，决不把“说了”当“做了”、把“做了”当“做完了”、把“做完了”当“做好了”。认真落实省市《关于进一步规范会议活动的通知》精神，从严控制会议数量、规模、规格和时间，强力整治文山会海。优化督查考核方式，从严控制监督检查的总量和频次，切实解决重“痕”不重“绩”、留“迹”不留“心”等问题，把基层从提供材料的忙乱中解放出来，把干部从无谓的事务中解放出来，把领导从有名无实的会议中解放出来，让广大干部的更多精力集中到干事创业上来！

　　坚持清正廉洁做表率。重廉洁、守底线是提高政府执行力的基本保证。严格落实政府系统全面从严治党主体责任，深化“三同步”责任区工作制度，认真履行“一岗双责”。深入贯彻落实中央八项规定精神和省委市委实施意见，大力整治“四风”，树立“严紧硬实”新风。加强法治政府建设，推进依法行政。严格遵守行政决策程序规定，完善规范性文件合法性审核机制，努力提高依法科学民主决策水平。着力构建亲清新型政商关系，加强工程建设、土地出让、政府采购、产权交易等重点领域监管，强化审计监督，坚决纠正损害群众利益的不正之风，坚决查处各类违法违纪行为和腐败问题，切实做到惩治力度决不减弱、零容忍态度决不改变、廉洁从政要求决不放松！

　　各位代表！时代是出卷人，我们是答卷人，人民是阅卷人。让我们更加紧密团结在以习近平同志为核心的党中央周围，在省委省政府和市委坚强领导下，不忘初心、牢记使命，凝心聚力、砥砺奋进，加快实现“两个走在前列”目标，努力交出一份让人民满意的答卷！
